# Investigating the cortical regions involved in MEP modulation in tDCS

**DOI:** 10.3389/fncel.2015.00405

**Published:** 2015-10-13

**Authors:** Ricardo Salvador, Cornelia Wenger, Pedro C. Miranda

**Affiliations:** Department of Physics, Faculdade de Ciências, Institute of Biophysics and Biomedical Engineering, Universidade de LisboaLisboa, Portugal

**Keywords:** transcranial magnetic stimulation, TMS, transcranial direct current stimulation, tDCS, motor cortex, finite element modeling, motor evoked potential

## Abstract

Transcranial magnetic stimulation (TMS) is used in several studies to evaluate cortical excitability changes induced by transcranial direct current stimulation (tDCS) of the primary motor cortex. Interpretation of these results, however, is hindered by the very different spatial distribution of the electric field (E-field) induced by the two techniques and by the different target neurons that they might act upon. In this study we used the finite element method to calculate the E-field distribution induced by TMS and tDCS in a realistically shaped model of a human head. A model of a commercially available figure-8 coil was placed over a position above the identified hand knob (HK) region. We also modeled two configurations of bipolar tDCS montages with one of the electrodes placed over the HK and a return electrode over the contralateral orbital region. The electrodes over the HK were either rectangular in shape, with an area of 35 cm^2^ or cylindrical with an area of π cm^2^ (1 cm radius). To compare the E-field distribution in TMS and the two tDCS models, average values of the E-field's magnitude as well as the polar and azimuthal angle were investigated in the HK region and premotor areas. The results show that both techniques induce fields with different magnitudes and directions in the HK: the field in tDCS is predominantly perpendicular to the cortical surface, contrary to what happens in TMS where the field is mostly parallel to it. In the premotor areas, the magnitude of the E-field induced in TMS was well below the accepted threshold for MEP generation, 100 V/m. In tDCS, the magnitude of the field in these areas was comparable to that induced at the HK with a significant component perpendicular to the cortical surface. These results indicate that tDCS and TMS target preferentially different neuronal structures at the HK. Besides, they show that premotor areas may play a role in the tDCS-induced after effects on motor cortex excitability.

## Introduction

Transcranial magnetic stimulation (TMS) is used in a number of studies to evaluate cortical excitability changes induced by transcranial direct current stimulation (tDCS; Nitsche et al., 2008). One cortical area studied extensively is the primary motor cortex (M1; Nitsche and Paulus, 2000). In these studies an initial assessment of the optimal position for stimulation of M1 is performed with TMS and then the average value of the magnitude of the motor evoked potential (MEP) in a target muscle is determined. After this stage, tDCS is applied at the same position for a given period of time, after which the average MEP size following TMS is again measured to determine changes relative to baseline. The results show that anodal tDCS of the M1 area results in an increased MEP size and that cathodal stimulation has the opposite effect (Nitsche and Paulus, 2000, 2001). The interpretation of these results, however, is not clear since the properties of the E-field induced by both techniques and, therefore, the mechanisms through each they act upon neurons are very different. The long-lasting (10–20 min) and low intensity (1–2 mA) currents injected through the electrodes during tDCS bring about subthreshold membrane polarizations of the cellular body of pyramidal neurons in the cortex which cause synaptic plasticity changes (Liebetanz et al., 2002; Nitsche et al., 2003; Radman et al., 2009). The E-field induced during TMS has very distinct properties, since it has a much shorter duration (hundreds of microseconds) and a much higher magnitude (~100 V/m; Roth et al., 1991; Kammer et al., 2001). It is thought to induce suprathreshold polarizations at bends and terminations of neurons in the cortex, provided they are correctly aligned with the applied E-field (Amassian et al., 1992; Nagarajan et al., 1993; Roth, 1994; Salvador et al., 2011).

A proper analysis of the results of this protocol requires, therefore, an accurate knowledge about the distribution of the E-field induced in the brain during both techniques. Since no direct *in vivo* measurements of the E-field in humans are possible, numerical techniques continue to be the only available means to predict the E-field distribution. Over the last few years several studies have been published describing numerical calculations of E-field distribution in realistic head models in both tDCS (Datta et al., 2009; Metwally et al., 2012; Miranda et al., 2013; Opitz et al., 2015) and TMS (Chen and Mogul, 2009; Opitz et al., 2011, 2013; Thielscher et al., 2011). However, to the best of our knowledge, no study has been conducted in which the field distribution in both techniques has been compared in the same head model. In this study we used the finite element (FE) method to numerically calculate the E-field induced in a realistic head model, built from magnetic resonance (MR) images of a single subject, during TMS and tDCS. In the analysis of the E-field in the cortex, we focused not only on the E-field's magnitude, but also on its direction. The results help shed light on the cortical areas targeted preferentially by both forms of stimulation.

## Materials and methods

### Head model

**Figure 1:**
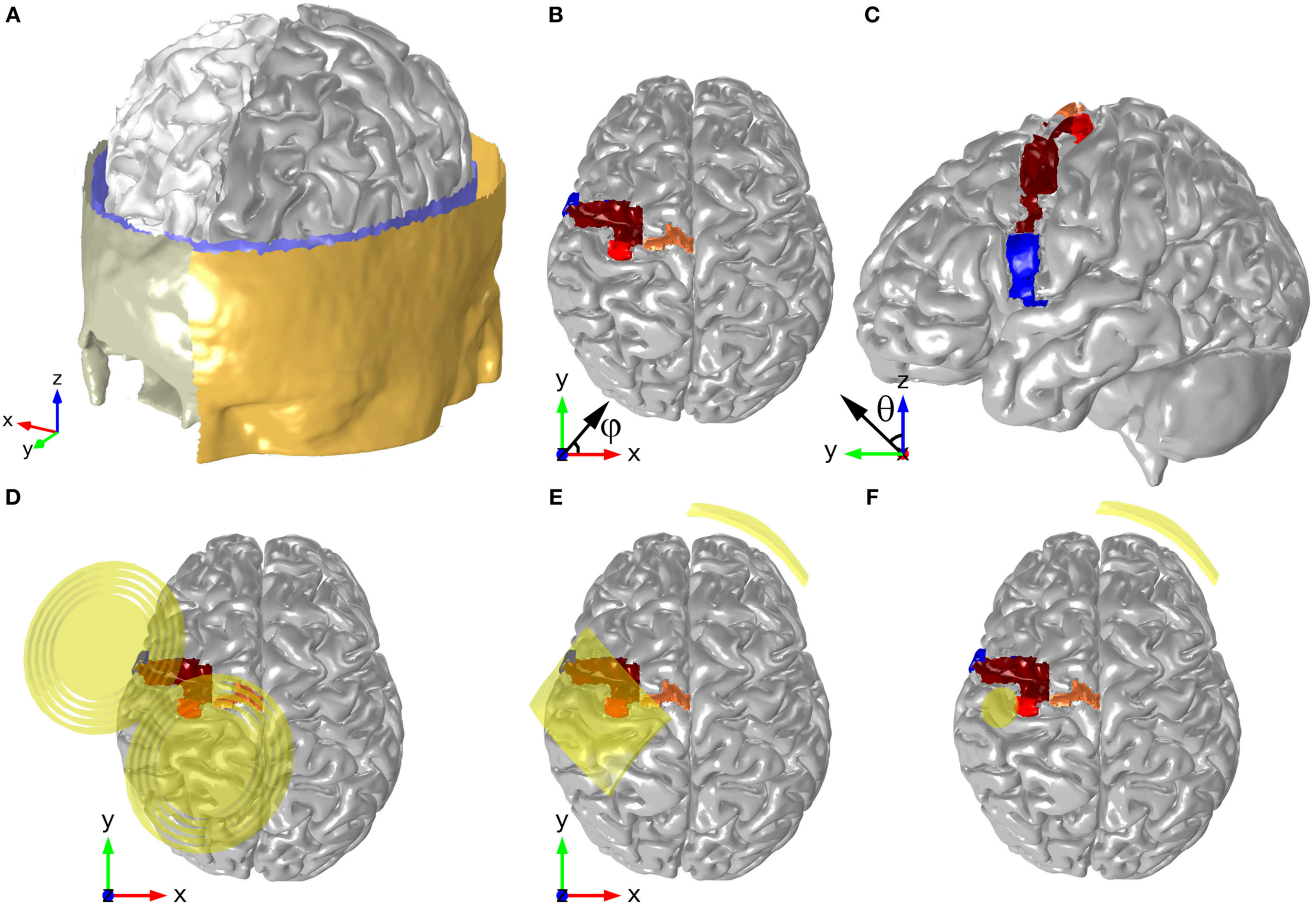
**Head model geometry, depicting the surfaces of the scalp, skull, CSF, GM, and WM (A)**. The position of motor and pre-motor areas are shown in panels **(B,C)**: HK (red), SMA (orange), PMd (brown), and PMv (blue). The positions and orientations of the TMS coil, the electrodes in the montage with two 35 cm^2^ electrodes and the ones in the montage with the π cm^2^ anode are shown in panels **(D–F)**, respectively. The angles shown in panels **(B,C)** represent the azimuthal angle (φ) and the polar angle (θ), respectively.

A realistic head model was created from T_1_- and T_2_-weighted images (1 mm^3^ isotropic resolution) of a 20 years old healthy female subject who agreed to participate in this study. The study was approved by the local ethical committee of Hospital Lusíadas, Lisbon, Portugal, where the images were obtained. Diffusion weighted images (DWI) were also acquired for the same subject (1.25 × 1.25 × 3.5 mm resolution). The images were registered to MNI space and surface meshes of the different tissues were obtained from the anatomical images using an adapted version of the SimNibs pipeline (http://www.simnibs.de), and the software package Brainsuite (http://brainsuite.org). These surface meshes were then corrected with the software Mimics (v16.0, www.materialise.com), which was also used to generate tetrahedral volume meshes suitable for FE analysis. The resulting meshes were free of irregularities such as holes in the CSF, and regions where the skull touched the GM outer surface. The meshes have more than 3.5 million tetrahedral second order elements. The quality of the mesh in Comsol is quantified by the parameter element quality which varies between 0 (degenerated low quality element) and 1 (best possible element; Comsol, 2013a). The meshes used in this study had an average element quality >0.4. The surface meshes for the different tissues are shown in Figure 1A.

### Geometry and placement of the electrodes and coils

The head model was prepared for tDCS E-field calculations by adding two electrodes on the scalp (see Figures 1E,F). Two different bipolar electrode configurations were considered, both with the anode—a rectangular 5 × 7 cm^2^ electrode or a circular one with a radius of 1 cm (π cm^2^)—centered over the left hand-knob. The latter was identified based on anatomical landmarks, as described by Yousry et al. (1997). The position of the hand-knob in the model is shown in Figures 1B,C. The rectangular electrode was tilted 45° from the anterior-posterior axis, which made its longer side approximately parallel to the central sulcus. The cathode in both cases was a 5 × 7 cm^2^ electrode placed over the contralateral eyebrow. The inner surface of all electrodes followed perfectly the curvature of the scalp, modeling a perfect electrode-scalp contact. All electrodes were modeled as homogeneous conducting media with a conductivity of 2 S/m, as was done in previous studies (Miranda et al., 2013). For the TMS calculations, the coil was also centered over the left hand-knob (Figure 1D). The coil replicates Magstim's 70 mm figure-8 coil, which has nine windings and a 90 mm separation between wing centers (Thielscher and Kammer, 2002). The coil's wires were modeled as circular lines placed at a distance of 9 mm from the scalp. In accordance with usual practice, the plane of the coil was tangential to the scalp surface and its handle was rotated by 45°from the anterior-posterior axis (Kammer et al., 2001).

### E-field calculation

All E-field calculations were performed using Comsol (v4.3b, www.comsol.com). To calculate the E-field distribution in tDCS, Laplace's equation subject to appropriate boundary conditions was solved to obtain the distribution of the scalar electric potential (ϕ) in the head model:

$$\vec{\nabla }\cdot \left(\sigma \vec{\nabla }\phi \right)=0$$

where σ is the electrical conductivity tensor. The E-field was then obtained taking the gradient of the scalar potential:

$$\vec{E}=-\vec{\nabla }\phi$$

By default Comsol enforces the continuity of the normal component of the current density at the inner boundaries of the model, and an electrical insulation boundary condition at the outer surfaces. We additionally imposed a floating potential boundary condition at the outer surface of each electrode (excluding the lateral surfaces). This boundary condition automatically sets uniform voltages at the outer surfaces of both electrodes so that the surface integral of the component of the current density perpendicular to electrode's surface is equal to a user specified value: 1 mA for the anode and –1 mA for the cathode.

The calculation of the field induced in TMS was slightly more complex since the E-field induced in this technique is actually the sum of two components: a primary component which only depends on coil geometry and a secondary component which depends also on the geometry of the head and its dielectric properties. These E-field components can be calculated from a magnetic vector potential $\left(\vec{A}\right)$ and the scalar electric potential (ϕ):

$$\vec{E}={\vec{E}}_{Pri}+{\vec{E}}_{Sec}=-\frac{d\vec{A}}{dt}-\vec{\nabla }\phi$$

Calculation of the primary component was based on the analytical solution for the E-field induced by circular coils with idealized “line” wires, as described in a previous study (Tofts, 1990):

$$A_{\varphi }=\frac{\mu_{0}I}{\pi k}{\left(\frac{a}{\rho }\right)}^{1/2}\left[K\left(k^{2}\right)\left(1-\frac{1}{2}\times k^{2}\right)-E(k^{2})\right]$$

where *A*_φ_ is the azimuthal component of the magnetic potential, $\mu_{0}=4\pi \times 10^{-7}\;H∕m$ is the magnetic permeability, *I* is the current in the coil, *a* is the coil's radius, ρ is the distance between the point where *A*_φ_ is being calculated and the coil's axis, *k*^2^ = 4*a*ρ[(*a* + ρ)^2^ + *z*^2^]^−1^ and *K* and *E* are elliptic integrals of the first and second kind, respectively. The primary E-field induced by the figure-8 coil modeled in this work was then obtained by summing the individual contribution of each circular coil composing it. These analytical solutions were implemented in Matlab (v2013b, www.mathworks.com). This information was then imported into Comsol as a source term in Laplace's equation, which was used to calculate the secondary component of the E-field:

$$\vec{\nabla }\cdot \left(\left(\sigma +jw\varepsilon_{0}\varepsilon_{r}\right)\vec{\nabla }\phi +\sigma \frac{d\vec{A}}{dt}\right)=0$$

where $\varepsilon_{0}=8.8542\times 10^{-12}F∕m$ is the vacuum permittivity, ε_*r*_ is the relative permittivity and *w*, the angular frequency is equal to 2π*f* where *f* is the frequency of the current in the coil. The same boundary conditions that were discussed earlier for the tDCS calculation were also imposed to the inner and outer boundaries of this model. We assumed that the current in the coil varied sinusoidally with a frequency of 5 kHz and a peak amplitude of 2133 A, which yields a maximum value of d*I*/dt of 67 A/μs. This corresponds to the average resting motor threshold (RMT) for this coil (Kammer et al., 2001).

The isotropic conductivity values of the tissues for both models were based on the ones used in previous studies (Miranda et al., 2013): 0.33 S/m for the scalp and gray matter (GM), 0.008 S/m for the skull, 1.79 S/m for the cerebrospinal fluid (CSF), and 0.15 S/m for the white matter (WM). E-field calculations were also performed taking into account the anisotropic conductivity of the WM and GM, as determined from DWI data. The latter were processed with the volume-normalized mapping approach of the SimNibs pipeline (Opitz et al., 2011) to find the conductivity values from the diffusion tensor components. In agreement with other studies (Thielscher et al., 2011), the relative dielectric permittivity of all tissues was set to one in the TMS model, which is equivalent to ignoring any capacitive effects of tissues in the calculation of the E-field (Roth et al., 1991). This approximation is considered valid for frequencies typical of TMS pulses (Plonsey and Heppner, 1967).

For the tDCS calculation, an iterative solver was used (BiCGStab) with the preconditioner Geometric Multigrid. The relative tolerance parameter, that determines when the solution has converged, was set at 10^−3^. For the TMS model, we used a different iterative solver (GMRES) with the preconditioner Incomplete LU (set with a drop-tolerance of 5 × 10^−4^). The same relative tolerance of 10^−3^ was used in this calculation. For more details on these solvers the interested reader may refer to Comsol (2013b).

## Results

In order to analyze and compare the E-field distribution induced in the different configurations modeled in this study, we calculated average volume values for: the E-field's magnitude, the azimuthal angle (the angle between the projection of the E-field onto the axial plane and the x-axis, i.e., the axis pointing from the left to the right hemisphere, Figure 1B) and the polar angle (the angle of the E-field with respect to the z-axis, i.e., the inferior-superior axis, Figure 1C). These average values were calculated in the GM in regions identified as important in stimulation of the hand motor area (see Figures 1B,C): hand-knob (HK), whole motor cortex (M1), supplementary motor area (SMA) and lateral premotor cortex (ventral, PMv, and dorsal, PMd). Identification of these landmarks was based on descriptions available in the literature about their shape, position and boundaries (Dum and Strick, 1991; Mayka et al., 2006). In summary, we considered that the lateral premotor cortex (PMv and PMd) ranged between the anterior part of the precentral gyrus and the precentral sulcus (posterior–anterior direction), and the lateral sulcus and the virtual continuation of the superior frontal sulcus (lateral–medial direction). The boundary between the PMv and PMd was considered to be the virtual continuation of the inferior frontal sulcus. The SMA ranged between the medial boundary of the PMd and the central fissure (lateral–medial direction) and its ventral boundary was defined as the cingulate sulcus. Due to difficulties in precisely selecting all these regions in the model, and the lack of knowledge about their precise anatomical boundaries, there is some error associated with their definition. However, some of these errors are minimized by presenting average values for the figures calculated in this study. The latter are expected to be less sensitive to misrepresentations of the regions of interest considered here (the interested reader may refer to supplementary material for more data supporting this).

### Electric field distribution in TMS

**Figure 2:**
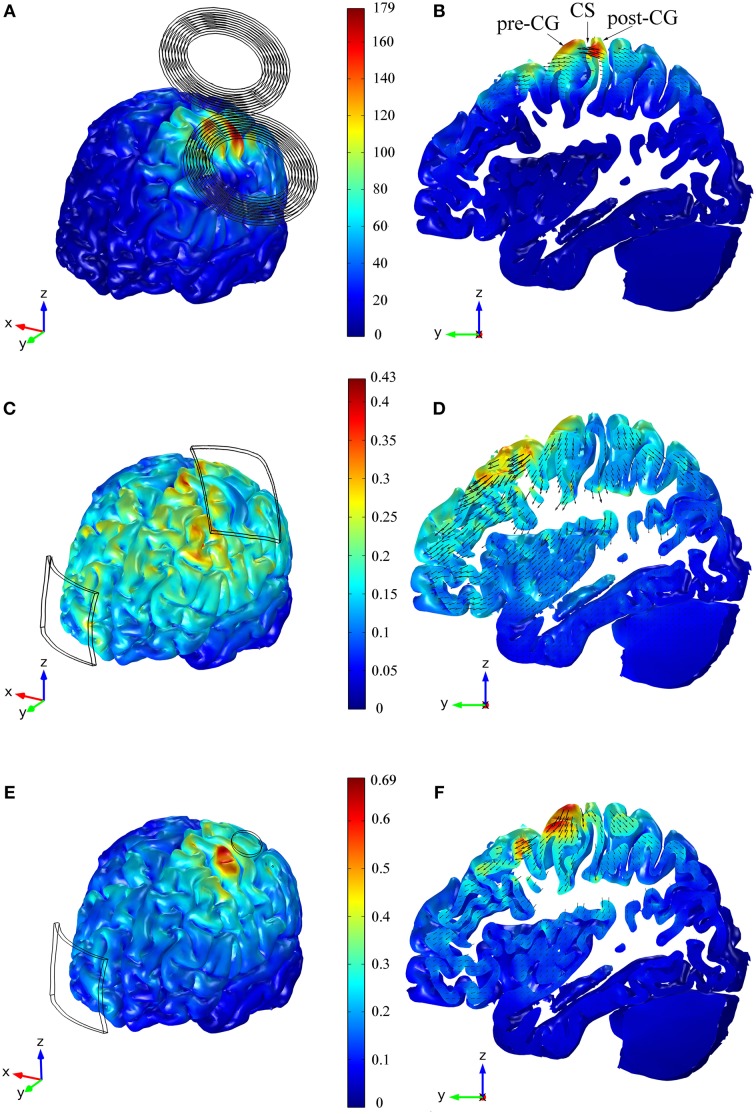
**Distribution of the E-field's magnitude induced during TMS (A,B), tDCS with the 35 cm^2^ electrode (C,D) and tDCS with the π cm^2^ anode (E,F)**. The figures in the left column show the E-field's magnitude in the GM volume, whereas the figures in the right column show the E-field's distribution in a sagittal view of a slab of GM tissue with a thickness of 1.3 cm and passing through the HK and PMd regions. The figures within the same row share the same color scale (E-field's magnitude in V/m). The figures in the right column also show vector plots of the projection of the E-field in the depicted sagittal plane. Panel **(B)** also shows the location of the central sulcus (CS), pre and post-central gyrus (pre-CG/post-CG).

The main feature of the distribution of the magnitude of the E-field was the presence of two maxima on the cortical surface: an absolute maximum in the post-central gyrus and a local maximum in the pre-central gyrus (see Figures 2A,B). In the anisotropic model, the E-field on the post- and pre-central gyri reached 177 and 144 V/m, respectively. The field's magnitude was strongest under the coil windings and its value rapidly decreased with distance from the coil. In the central sulcus near the HK, the field was stronger in the posterior than in the anterior wall, and it decreased rapidly with depth (see Figure 2B).

The E-field induced in TMS was very localized in the regions located under the coil's center (see Figure 2A). As such the E-field's magnitude average value was stronger in the HK than in any pre-motor area, as can be seen in Table 1. In the HK, the average azimuthal angle was 44° very similar to the orientation of the central section of the coil. Also in that region, the average polar angle was 77°. This suggests that the E-field induced in TMS is predominantly tangential to the cortical surface at the top of the pre-central gyrus, with only a small upward component, as is clearly shown in Figure 2B.

**Table 1:** **Average and maximum values of the E-field's magnitude, polar angle, azimuthal angle and angle between the field induced in tDCS and TMS (θ_***TMS***ˆ***tDCS***_) in the different regions of interest (ROIs)**.

**ROI**	**E-field's magnitude average|maximum (V/m)**						**Polar angle(°)**			**Azimuthal angle (°)**			**θ_*TMS*ˆ*tDCS*_(°)**	
	**TMS**		**35 cm**^**2**^		**π cm**^**2**^		**TMS**	**35 cm^2^**	**π cm^2^**	**TMS**	**35 cm^2^**	**π cm^2^**	**35 cm^2^**	**π cm^2^**
HK	65	144	0.16	0.29	0.34	0.74	76	129	136	44	13	17	62	68
SMA	33	93	0.17	0.39	0.22	0.53	87	120	117	33	24	33	36	33
PMd	41	146	0.19	0.36	0.22	0.69	85	128	129	53	39	53	52	53
PMv	16	47	0.15	0.25	0.15	0.26	92	139	138	107	52	126	123	126
C	7	19	0.15	0.37	0.15	0.38	149	100	100	74	52	53	91	91

*The labels 35 cm^2^ and π cm^2^ refer to the size of the anode in the different tDCS models. The labels of the ROIs have been identified previously in the text, except label C, which represents a circular regions located under the position of the cathode (A = 25 cm^*2*^)*.

Changing the position of the coil 1 cm anteriorly (along the line between the two electrodes) led to only small changes in the field distribution. The overall maximum was still located at the top of the post-central gyrus and its value decreased only slightly to 175 V/m, as compared to 179 V/m in the original model. The local maximum and the mean values over the HK also increased somewhat in the model with the translated coil: 10.6 and 8.3%, respectively (values are expressed as a percentage of the value in the original model). This slight increase was also observed in all the pre-motor regions.

### Electric field distribution in tDCS

In the tDCS model with the larger anode, the highest E-field values were found at the crowns of some of the gyri located closer to the electrodes' edges (see Figure 2C). At the bottom of the sulci under the electrodes there were also small regions with very high E-field magnitude. In the cortical regions between the two electrodes and away from their edges, the local maxima were located at the top of the gyri. However, the E-field's magnitude in these regions was smaller than in the former regions. Contrary to what happened in the TMS model, the E-field induced during tDCS (35 cm^2^ anode) was stronger on the crown of the pre-central gyrus (0.30 V/m) than on the crown of the post-central gyrus (0.10 V/m), as shown in Figure 2D. The global maximum of 0.43 V/m occurred in very localized regions located on the gyri near the edges of the electrodes and at the bottom of the sulci beneath it. The field was again low on the walls of the central sulcus but had a local maximum at the bottom of the central sulcus that is comparable to the value at the crown of the pre-central gyrus.

In the model with the smaller anode, the global maximum was located on the crown of the pre-central gyrus, as shown in Figure 2E. This again corresponds to the position of the edge of the electrode. There, the maximum of the E-field's magnitude was higher than the global maximum obtained in the model with the larger anode: 0.69 and 0.43 V/m, respectively. At the bottom of the central sulci under the electrode there was again a local maximum of the E-field's magnitude: 0.40 V/m (Figure 2F). At the top of the gyri between the two electrodes, away from the electrode's edges, the distribution of the E-field was similar in the two models. The E-field distribution under the cathode was also similar in both models, and this is reflected in similar average values in the row labeled C in Table 1.

The E-field induced during tDCS was less focal than the one induced during TMS, regardless of anode size. As a consequence, the average E-field's magnitude in pre-motor areas was comparable to the average E-field's magnitude in the HK, particularly for the 35 cm^2^ electrode (see Table 1). The orientation of the E-field in these two regions, however, was very different. The field was essentially perpendicular to the cortical surface on the crown of the gyri under the anode and cathode, whereas it was predominantly tangential to the gyri between electrodes (see Figures 2D,F). In the PMd, since this region was still mostly covered by the large 35 cm^2^ placed over the HK, this resulted in average polar angles comparable to those obtained in the HK (see Table 1). This indicates that the field has a strong component perpendicular to the surface of the gyri. In the model with the π cm^2^ anode, the mean polar angle at the PMd was slightly smaller than that over the HK. At the PMv, the opposite happened, with a higher mean polar angle than at the HK region. At the SMA, the mean polar angles were again lower than those over the HK which corresponds to an E-field with a weaker component perpendicular to the gyri in this region. The average azimuthal angle was low in the HK, indicating a strong left-right component. In the pre-motor areas, it increased to values closer to the azimuthal angle of the line defined by the two electrodes (24°–53°, as shown in Table 1). The exception occurred at the PMv in the model with the π cm^2^ anode, where the mean azimuthal angle was 126° indicating a stronger right-left component.

For the model with the π cm^2^ anode the average field in the HK increased by 112.5% relative to the model with the 35 cm^2^ electrode. The average field in the premotor areas did not change much with electrode size: in the model with the smaller electrode, the mean E-field increased 29.4% at the SMA and 15.8% at the PMd. At the PMv the average field was independent of electrode size. Regarding the variation of the average polar and azimuthal angles, they showed distinct behaviors: the average polar angle varied only slightly between the two models (maximum variation of 7° at the HK), whereas the average azimuthal angle increased significantly in all areas for the model with the smaller electrode (maximum variation of 74° at the PMv).

### Comparison between the field induced in tDCS and TMS

**Figure 3:**
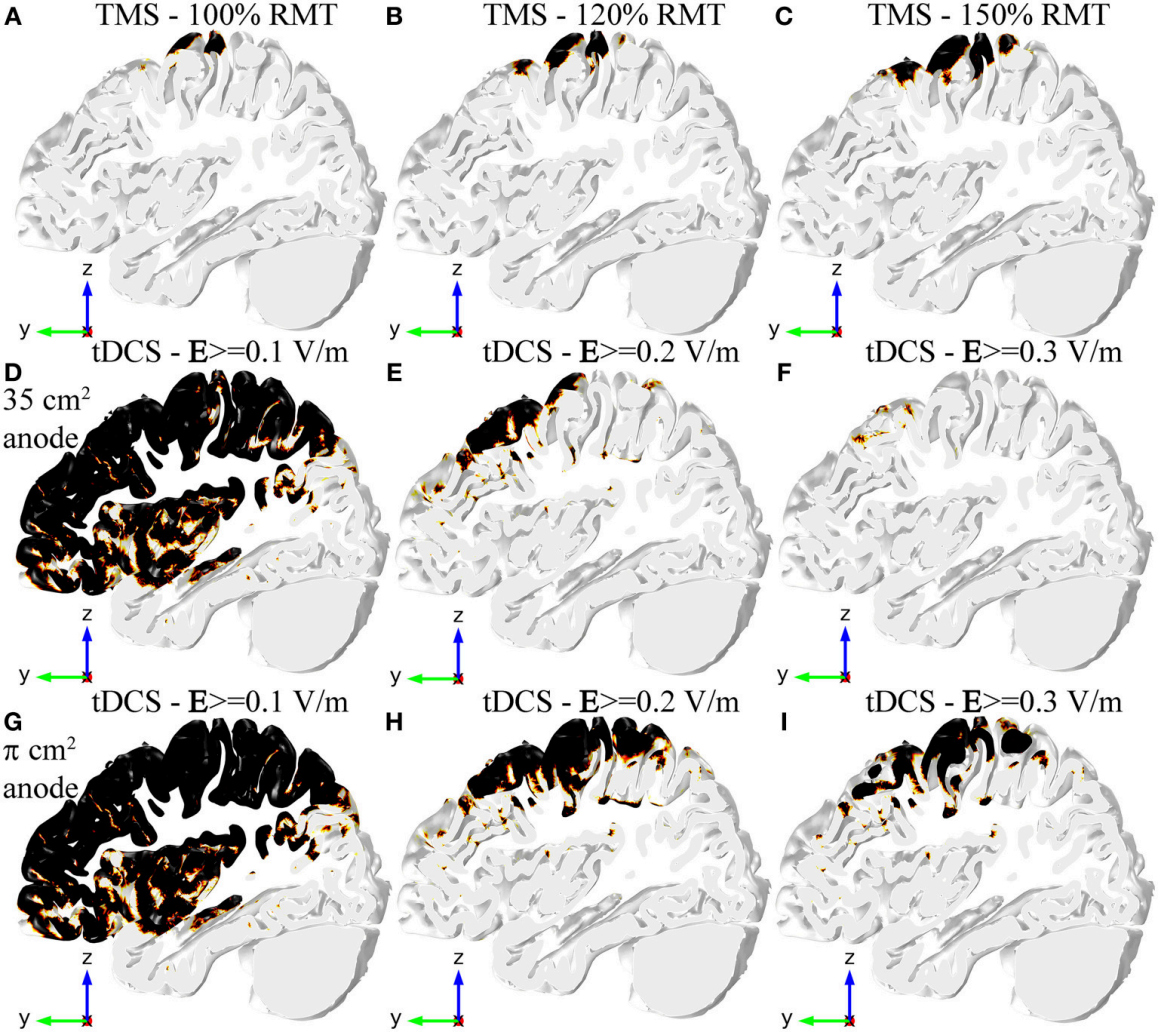
**Regions in the GM volume where the field is above a specified threshold in TMS (A–C), tDCS with the 35 cm^2^ electrode (D–F), and tDCS with the π cm^2^ anode (G–I)**. Regions shown in black are above threshold. The region selected is the same slab of GM tissue passing through the HK and PMd shown in Figure 2. The threshold for TMS was set to 100 V/m in all figures, but the stimulator output was set to 100% RMT **(A)**, 120% RMT **(B)** and 150% RMT **(C)**. The threshold for tDCS was set to 0.1 V/m **(D,G),** 0.2 V/m **(E,H)** and 0.3 V/m **(F,I)**.

In TMS an E-field magnitude of 100 V/m is a well-established value for the threshold for MEP generation, in good agreement with our previous work (Salvador et al., 2011). The only regions where the E-field magnitude was greater than this value were the crown and lip of the pre- and post-central gyri. Increasing the stimulator output made the E-field magnitude reach values above threshold in more gyri. The same thing happened for a greater portion of the posterior wall of the central sulcus (see Figures 3A–C). The field in the anterior wall of the central sulcus, however, only reached threshold values very close to maximum stimulator output for the Magstim 200 stimulator (data not shown).

In tDCS there is no threshold value described in the literature for the onset of the excitability changes induced by the E-field. Therefore, we considered several possible threshold values from 0.1 V/m to 0.3 V/m (Figures 3D–I). A threshold level of 0.1 V/m was attained in most regions under the electrodes and in between them, regardless of anode size (Figures 3D,G). Increasing the threshold value to 0.2 V/m decreased the regions above threshold substantially (see Figures 3E,H), especially in the model with the 35 cm^2^ anode (Figure 3E). In that model, the only regions above 0.2 V/m were the crown of the pre-central gyrus and gyri located anterior to it. The bottom of the central sulcus was also above threshold. In the model with the smaller anode the regions above this threshold extended further, now including also the posterior wall of the central sulcus and gyri located posterior to the central sulcus (including the post-central gyrus). Regions above a threshold value of 0.3 V/m were very localized in the model with the 35 cm^2^ anode (Figure 3F). In the model with the π cm^2^ anode, however, the regions above this threshold still occupied a great part of the pre-central gyrus and the posterior wall of the central sulcus, as well as the post-central gyrus and parts of gyri located anterior to the HK (Figure 3I).

From the description of the direction of the E-field induced in TMS and tDCS, it is expected that the mean angle between the E-field induced by these two techniques is high. That was indeed the case in all the cortical regions considered (see Table 1, last column), especially the HK, PMv and the circular regions located under the cathode (C, in Table 1). In the SMA and PMd, the mean angles were smaller.

### Influence of tissue anisotropy

**Figure 4:**
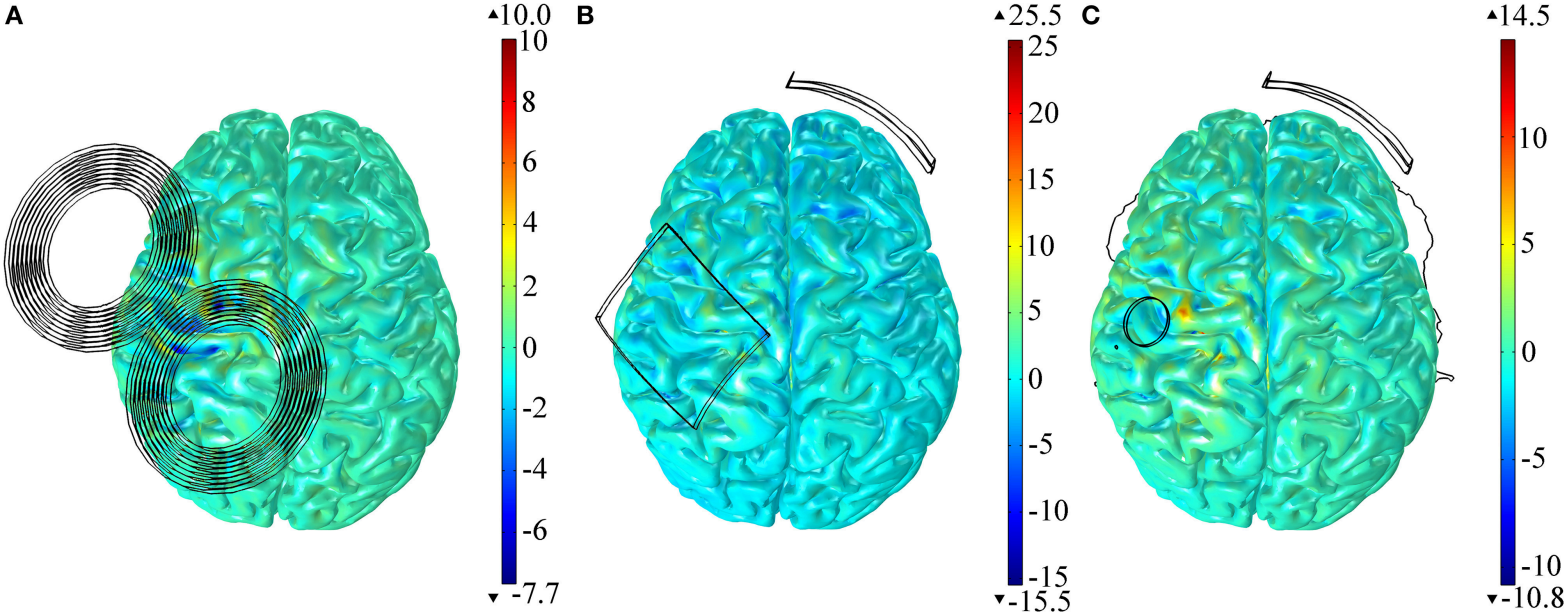
**Relative difference between the E-field magnitude distribution in the cortex in the anisotropic and the isotropic models for TMS (A), tDCS with the larger 35 cm^2^ anode (B) and tDCS with the π cm^2^ anode (C)**. All results are presented as a percentage of the maximum E-field induced in the cortex in the anisotropic model.

All the results presented before were obtained in the model with anisotropic conductivities in the GM and WM tissues. Modeling the tissues as isotropic, however, had little effect on the E-field distribution in both TMS and tDCS (regardless of electrode size). To quantify this we calculated the differences between the variables identified in Table 1 in the anisotropic models and the isotropic ones. As before, we performed this calculation for all the areas identified. The results, presented as a percentage of the values obtained in the anisotropic model, show that the maximum difference was of only +3.9% (azimuthal angle in the SMA region for the model with the 35 cm^2^ electrode over M1) and the minimum difference was of −0.2% (E-field's magnitude in the PMv region, for the tDCS model with the largest electrode over M1). The spatial distribution of the relative difference of the E-field's norm is shown in Figure 4 for both the TMS and tDCS models. The results indicate that effects of anisotropy, albeit always small, are larger in the tDCS models than in the TMS one.

## Discussion

### Differences in E-field distribution in TMS and tDCS

This study provides important insights into the E-field distribution in TMS and tDCS and how it affects neurons in the cortex. The most prominent feature of the E-field induced in TMS was the presence of two maxima at the top of the pre- and post-central gyri. Moving the coil anteriorly proved inefficient at decreasing the maximum over the post-central gyrus significantly. These results are consistent with what was found in other studies reporting that when the primary E-field (which depends only on coil orientation and geometry) is perpendicular to the local orientation of the gyrus, the total E-field is strongest at the gyrus (Thielscher et al., 2011). They also indicate that projections from the somato-sensory cortex to the primary motor cortex may be stimulated in response to the TMS pulse. The latter have been suggested to play an important role in the results of TMS of the primary motor cortex (Esser et al., 2005; Salvador et al., 2011). Another important characteristic of the induced field is that it does not extend significantly along the depth of the anterior wall of the central sulcus, even with increasing stimulator output. Also, the induced field is mostly parallel to the cortical surface at the top of the gyri, and perpendicular to it in the sulci. Since only neuronal processes parallel to the field are affected by it (Roth, 1994), it is unlikely that the TMS-induced field directly stimulates pyramidal neurons at the top of the gyri, given that these are mostly oriented perpendicularly to the cortical surface (Kammer et al., 2007). In principle, stimulation could take place in the walls of the sulci, since there the field is aligned properly with pyramidal cells. However, the magnitude of the induced E-field on the sulcal walls decreases rapidly with depth so stimulation may occur only at stimulator outputs higher than RMT. The E-field at the top of the gyri will, however, have a strong effect on the cortical interneurons aligned mostly tangentially to the cortical surface and on collaterals of pyramidal cells with the same orientation (Nagarajan et al., 1993; Salvador et al., 2011). These structures, when aligned tangentially to the induced E-field, can be strongly polarized at their terminations.

The E-field induced in tDCS has very distinct characteristics to the one induced in TMS. One of these differences is its magnitude: the E-field's magnitude in tDCS is two to three orders of magnitude smaller than that of the field induced in TMS. Also, the location of the maxima of the E-field's magnitude is very different between the two techniques. Whereas in TMS the maxima are located at the top of the two gyri closest to the coil center, in tDCS with the 35 cm^2^ anode there is only one local maximum at the top of the pre-central gyrus, together with global maxima located at the crowns of the gyri closest to the anterior edges of the anode and at the bottom of the sulci under the electrodes. This suggests that this tDCS configuration may target not only motor but also pre-motor areas, as illustrated by the relatively high E-field values in these regions. The field induced in TMS, on the other hand, is much more localized in areas closer to the primary motor cortex. In the PMd there is also a strong maximum of the E-field induced in TMS, but its average magnitude is only 63% of the average over the HK region. In the tDCS model with the π cm^2^ electrode, the global maximum shifts to the crown of the pre-central gyrus, due to the fact that the edge of the electrode is located above it. For this electrode configuration, in the pre-motor areas, the average E-field magnitude values are always smaller than those obtained at the HK.

Another important difference between the two field distributions is related to the orientation of the E-field. In the motor cortex, the orientation of the E-field induced by these two techniques is remarkably different, with the E-field induced in tDCS (regardless of anode size and shape) having a component predominantly perpendicular to the cortical surface at the top of the gyri. This is shown by the mean values of the polar angle in the HK, which are higher in tDCS than in TMS. This indicates that tDCS probably targets preferentially neural processes perpendicular to the cortical surface at the top of the pre-central gyrus, such as pyramidal neurons. Since the latter have been described as the most likely target in tDCS (Radman et al., 2009), these results indicate that this electrode configuration is indeed capable of stimulating those cells in the HK. Contrary to TMS, in tDCS the E-field at the top of the post-central gyrus is always relatively low. However, this region may be targeted with the smaller anode if the threshold value for tDCS efficacy is set to a sufficiently low value (about 0.3 V/m in this study). In the pre-motor areas, the orientation of the E-field induced by both techniques tends to be slightly more similar (especially in the SMA) but big differences can still be found. This is particularly visible in the much bigger polar angles obtained in the PMd and PMv. In the PMd, the large mean polar angle indicates that the E-field might target the same type of neural processes mentioned before in the case of the HK, i.e., structures located perpendicularly to the surface of the gyri. The interpretation is harder for the PMv region, since this region is located laterally in the head. For this region it is the mean azimuthal angle that better predicts whether the field in perpendicular or not to the cortical surface at the top of the gyri. The mean azimuthal angle at the PMv in the model with the 35 cm^2^ electrode is 52° which indicates a strong left-right direction and, therefore, a strong inward component of the field perpendicular to the cortical surface at the top of the gyri. Interestingly, in the model with the π cm^2^ electrode the mean azimuthal angle is 126° which indicates a perpendicular component but pointing outwards from the cortical surface at the top of the gyri. These results indicate that the E-field induced in tDCS is capable of affecting pyramidal cells in these pre-motor regions as well. In the SMA the E-field tends to be more parallel to the one induced in TMS which may indicate that in this region it is harder for tDCS to polarize the pyramidal cells perpendicular to the top of the gyral surface. Since several projections exist between pre-motor areas and the primary motor cortex (Dum and Strick, 1991), these results suggest that the PMd and PMv may be involved in the tDCS induced after-effects in motor cortex excitability.

### Effects of tissue conductivities and other model limitations

The bulk of the results presented in this work were obtained from anisotropic models of tissue conductivity. Our analysis, however, showed no substantial differences in E-field distribution between isotropic and anisotropic tissue conductivity models. This may stem from the fact that this analysis is based on E-field values in the GM and that the GM is mostly isotropic. This insensitivity to tissue anisotropy is in line with what was reported in a previous study (Opitz et al., 2011). In this work all the data analysis was presented in the GM because all neuronal processes affected by the E-field are located there. The only exceptions are the bends of pyramidal cells as they enter into the WM, which have been shown to be a possible excitation site in TMS of the motor cortex (Amassian et al., 1992; Maccabee et al., 1998). However, it is expected that the polarization at the bends depends strongly on the E-field magnitude along the direction of the neuron in the GM (Roth, 1994). Since the WM has a much higher fractional anisotropy it is expected that the influence of anisotropy in E-field values there is higher.

Other studies point out the substantial effect of the conductivity of the skull in the E-field distribution during tDCS (Metwally et al., 2012; Opitz et al., 2015). In TMS it is expected that the influence of skull conductivity will have a small effect on the overall distribution of the E-field (Roth et al., 1991). Nonetheless, future tDCS work should focus on determining the effects of assigning different conductivity values to this tissue.

The way the stimulation electrodes were modeled in this work assumes that they are homogeneous conductive materials with a conductivity of 2 S/m. The conductivity of the material of the electrodes has been shown to have a negligible effect on the E-field distribution in the brain within the reasonable conductivity range of 0.2–20 S/m (Opitz et al., 2015). The fact that the contact between the electrode and the skin is modeled as perfect is more likely to affect the prediction of the induced E-field, particularly in the scalp. The particular way in which the coil was modeled in the TMS study has also been shown to not be accurate for regions very close to the coil wires (Salinas et al., 2007). We expect, however, that these effects are not very pronounced for the E-field distribution in the brain.

Another important limitation of this work is that only one head model was created. The question of inter-subject variability in this experimental protocol has been demonstrated lately in some works (López-Alonso et al., 2014; Wiethoff et al., 2014). This shows the importance of considering different head models since anatomical changes, like layer thickness and gyri variability, can have a strong effect on the E-field distribution (Laakso et al., 2015; Opitz et al., 2015). This is clearly shown by the differences between overall E-field distribution between this head model and the one presented in our previous work (Miranda et al., 2013). In that work, the E-field distribution presented global maxima at the bottom of the sulci beneath the electrodes. In the present work, only local maxima are found there, and the E-field global maxima are found at the top of the gyri. This is more strongly seen in the model with the smaller π cm^2^ electrode over the HK. This is most likely related to the thickness of the skull and CSF being smaller in this model as compared to the one in the last study. Regarding the effects of the variability of the cortical surface geometry, it is expected that it has a stronger effect in the TMS E-field calculation, since charge accumulation at the GM-CSF interface seems to explain the patterns of maxima observable in the E-field induced during this type of stimulation (Thielscher et al., 2011).

Future work should focus on the determination of how the E-field distribution affects the membrane potential of multicompartmental neuron models. Some studies do exist featuring such models, but often they assume either a simplified neuronal geometry (Salvador et al., 2011) or E-fields applied along the neuron that were not obtained from realistic volume conductor models (Kamitani et al., 2001; Wu et al., 2013). Models including both a realistic representation of the neuronal geometry, and a calculation of the E-field in a realistic volume conductor model are, to the best of our knowledge, inexistent for tDCS/TMS. Combining the latter with models of large scale assemblies of neurons (Esser et al., 2005; Merlet et al., 2013; Molaee-Ardekani et al., 2013) should lead to further insights on the mechanisms of TMS and tDCS.

## Funding

IBEB is funded by the Portuguese Foundation for Science and Technology (FCT), under project UID/BIO/00645/2013. CW is supported by Novocure.

### Conflict of interest statement

The authors declare that the research was conducted in the absence of any commercial or financial relationships that could be construed as a potential conflict of interest.
