# Supplementary material for: Investigating the cortical regions involved in MEP modulation in tDCS
Source: Front Cell Neurosci. 2015 Oct 13;9:405. doi: 10.3389/fncel.2015.00405 (PMC4602135; doi:10.3389/fncel.2015.00405)
Supplement: Supplementary file 1 [file Table1.DOCX]

Average E-field magnitude ($\bar{\left\| \vec{E} \right\|}$), azimuthal ($\bar{\varphi}$) and polar ($\bar{\theta}$) angle in a cortical volume defined by intersecting the entire GM volume with a cylinder of a specified radius (0.5 – 2.0 cm) centered over the HK and oriented perpendicularly to the cortex in that region. All average values were calculated for the TMS model and for both tDCS models (one with the 35 cm^2^ electrode and another with the$\pi$ cm^2^ electrode).

| Model | Radius | | | | | | | | | | | |
| --- | --- | --- | --- | --- | --- | --- | --- | --- | --- | --- | --- | --- |
|  | 0.5 cm | | | 1.0 cm | | | 1.5 cm | | | 2.0 cm | | |
|  | $\bar{\left\Vert\vec{E} \right\Vert}$  $(\frac{V}{m})$ | $\bar{\varphi} (^{\circ})$ | $\bar{\theta} (^{\circ})$ | $\bar{\left\Vert\vec{E} \right\Vert}$  $(\frac{V}{m})$ | $\bar{\varphi} (^{\circ})$ | $\bar{\theta} (^{\circ})$ | $\bar{\left\Vert\vec{E} \right\Vert}$  $(\frac{V}{m})$ | $\bar{\varphi} (^{\circ})$ | $\bar{\theta} (^{\circ})$ | $\bar{\left\Vert\vec{E} \right\Vert}$  $(\frac{V}{m})$ | $\bar{\varphi} (^{\circ})$ | $\bar{\theta} (^{\circ})$ |
| TMS | 56,4 | 53,5 | 147 | 55,7 | 55,9 | 147 | 53,6 | 56,4 | 145 | 49,5 | 56,9 | 144 |
| 35 cm^2^ | 0,13 | 13,3 | 134 | 0,14 | 14,4 | 136 | 0,14 | 18,2 | 135 | 0,14 | 21,0 | 135 |
| $\pi$ cm^2^ | 0,24 | 20,9 | 69,9 | 0,24 | 27,1 | 70,6 | 0,24 | 33,1 | 68,8 | 0,23 | 38,1 | 66,4 |
